# Supplementary material for: Association between oral microbiome and seven types of cancers in East Asian population: a two-sample Mendelian randomization analysis
Source: Front Mol Biosci. 2023 Nov 21;10:1327893. doi: 10.3389/fmolb.2023.1327893 (PMC10702768; doi:10.3389/fmolb.2023.1327893)
Supplement: Supplementary file 1 [file DataSheet1.ZIP › Supplementary Materials/MR plots for tongue/tongue═╝/Ovarian cancer/pheno.1803_to_ovarian cancer_leave_one_out.pdf]

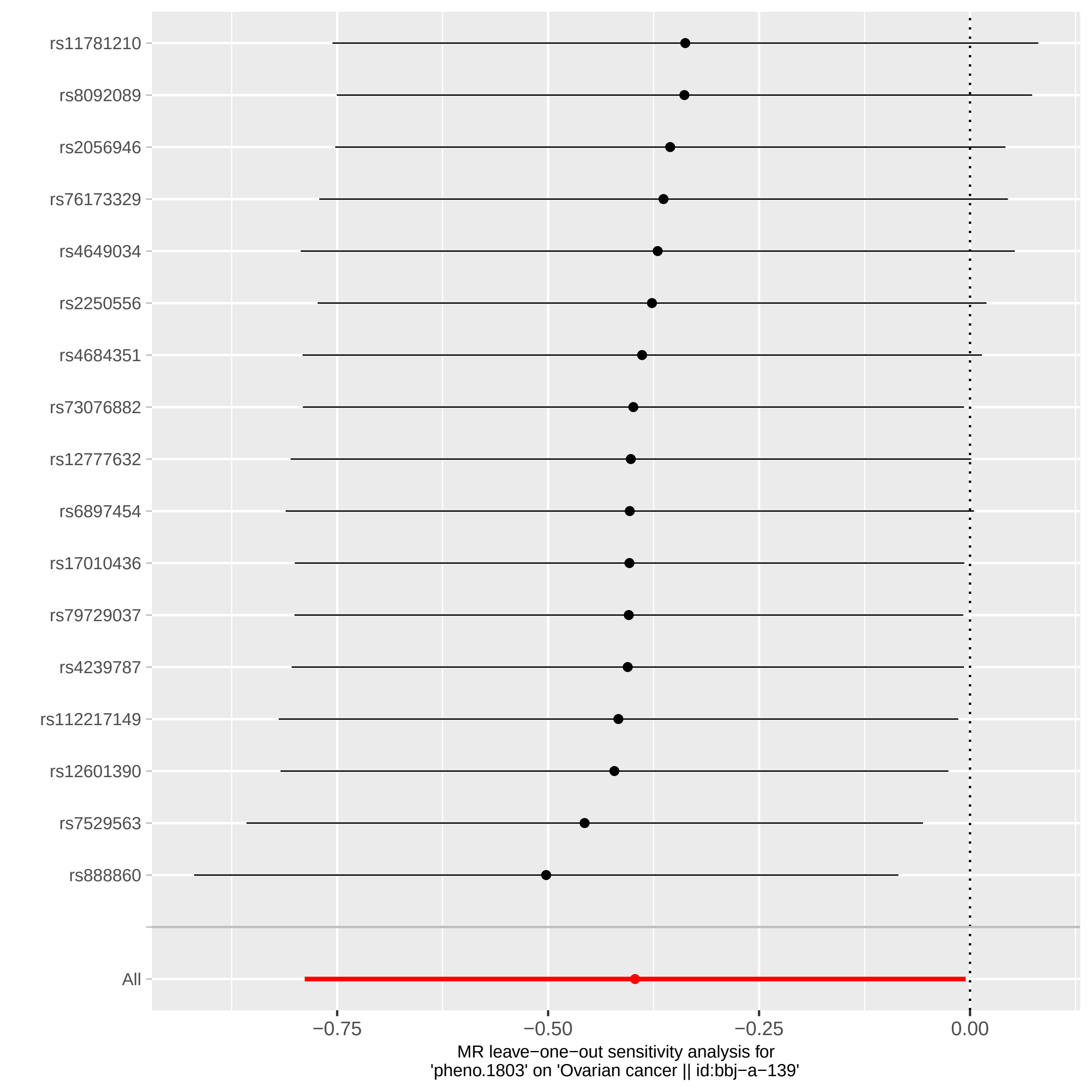
rs11781210
rs8092089
rs2056946
rs76173329
rs4649034
rs2250556
rs4684351
rs73076882
rs12777632
rs6897454
rs17010436
rs79729037
rs4239787
rs112217149
rs12601390
rs7529563
rs888860
All
−0.75
−0.50
−0.25
0.00
MR leave−one−out sensitivity analysis for
'pheno.1803' on 'Ovarian cancer || id:bbj−a−139'
